# Analysis of hippocampal local field potentials by diffusion mapped delay coordinates

**DOI:** 10.1007/s10827-024-00870-6

**Published:** 2024-04-06

**Authors:** D. A. Gonzalez, J. H. Peel, T. Pagadala, D. G. McHail, J. R. Cressman, T. C. Dumas

**Affiliations:** 1 Interdisciplinary Program in Neuroscience, George Mason University, Fairfax, VA 22030 USA; 2 Psychology Department, George Mason University, 4400 University Drive, MS 2A1, Fairfax, VA 22030 USA; 3 Department of Physics and Astronomy, George Mason University, Fairfax, VA 22030 USA

**Keywords:** Hippocampus, Local field potential, Diffusion mapped delay coordinates

## Abstract

Spatial navigation through novel spaces and to known goal locations recruits multiple integrated structures in the mammalian brain. Within this extended network, the hippocampus enables formation and retrieval of cognitive spatial maps and contributes to decision making at choice points. Exploration and navigation to known goal locations produce synchronous activity of hippocampal neurons resulting in rhythmic oscillation events in local networks. Power of specific oscillatory frequencies and numbers of these events recorded in local field potentials correlate with distinct cognitive aspects of spatial navigation. Typically, oscillatory power in brain circuits is analyzed with Fourier transforms or short-time Fourier methods, which involve assumptions about the signal that are likely not true and fail to succinctly capture potentially informative features. To avoid such assumptions, we applied a method that combines manifold discovery techniques with dynamical systems theory, namely diffusion maps and Takens’ time-delay embedding theory, that avoids limitations seen in traditional methods. This method, called diffusion mapped delay coordinates (DMDC), when applied to hippocampal signals recorded from juvenile rats freely navigating a Y-maze, replicates some outcomes seen with standard approaches and identifies age differences in dynamic states that traditional analyses are unable to detect. Thus, DMDC may serve as a suitable complement to more traditional analyses of LFPs recorded from behaving subjects that may enhance information yield.

## Introduction

Synchronous activity amongst large populations of neurons in the hippocampus creates rhythmic oscillations that coordinate the discharge activity of neuronal ensembles. The most well studied hippocampal oscillations observed in local field potentials (LFPs) occur between 6–12 Hz (theta), 25–55 Hz (slow gamma), and 60–100 Hz (fast gamma) along with broad spectral 200 Hz LFP complexes known as sharp wave ripples (SWRs, that include a late 140–200 Hz ripple component). While informative, traditional frequency analyses may not be ideal for interrogation of hippocampal oscillations. For example, Fourier transforms used to quantify the power of selected frequencies residing within a complex signal operate by decomposing signals into collections of sinusoids. Since neuronal network dynamics are, in general, quasi-periodic and nonlinear by nature (Perrenoud & Cardin, 2023), Fourier transforms may exclude information by over-simplifying the signal and both quasi-periodicity and nonlinearity are hard to succinctly quantify with a Fourier transform. Moreover, methods to determine if an oscillatory event occurred and when a given oscillation event begins and ends can be somewhat arbitrary (McHail & Dumas, 2020; Segneri et al., 2020; Ventrucci et al., 2014).

To address these issues, we propose the use of diffusion mapped delay coordinates (DMDC). This DMDC analysis assumes that the dynamical state of the system is, for the time of the measurement, in a stable, or steady state. This may not be strictly true for all samples, but works well as a starting assumption for LFPs recorded at stationary locations within the hippocampus. Such steady states can be described by fixed points, periodic signals, and, more generally, trajectories in a potentially high dimensional space, sometimes referred to as attractors, or manifolds. DMDC operates under the assumption that each temporal measurement, which we take as 2500 ms, or 2500 samples, for this study, is a sample of a single dynamic manifold. The method is based on combining Takens’ time-delay embedding theory for chaotic systems and a technique called diffusion maps. If one were to observe all of the processes of a dynamical system, the trajectory tracing out the movement through the large space of these observations would, if deterministic, never cross itself. If the number of observations is limited to a subset of the relevant dynamics, as is typically the case, the observations will produce trajectories in their limited space that will almost certainly overlap. Takens’ theorem is a powerful tool that enables the disentanglement of these non-linear dynamics by casting the data into a higher dimensional space through the process of time-delay embedding. The new manifold, created by the time-delay embedding, is not the same as the full manifold of relevant dynamics, but Takens proved that they should have the same topology.

The second part of the DMDC algorithm, the diffusion maps algorithm, assumes that the density of points on the time-delayed manifold can be used to find the solutions to a presumed diffusive process on the manifold. When ordered by amplitude, the spectrum of eigenvalues of the diffusive process can provide insights into the geometric structure of the underlying dynamical manifold. In fact, the geometrical properties of a space can be inferred through the eigenvalue spectrum through another powerful mathematical relation called Weyl’s law that can be used to determine the geometric features, such as the dimension and volume of the manifold. The dimensionality of a manifold is a rough measure of its independent degrees of freedom. In the case of a fixed point, the dimensionality is 0, a periodic orbit, 1, or a surface, 2. The dimensionality of a manifold will be N or lower in a general N dimensional space. For complex dynamics, fractal structures can form and the dimension can be non-integer. Volume is the higher dimensional analog to period, and loosely represents the length (in timesteps) of a quasi-period.

DMDC has been used successfully to model nonlinear and dynamic physical systems (Berry et al., 2013) but has yet to be applied to neural signals. To that purpose, we applied DMDC to LFP data previously analyzed using traditional frequency analyses (McHail & Dumas, 2020). Prior analyses found significant effects of developmental stage and a positive AMPA receptor modulator on slow gamma power and fast gamma event rate, peak power, and peak frequency. However, these effects were tied most to duration of time spent in the maze or movement speed, not location or any other behavioral effects. Here we report that DMDC reveals age effects for Dimension in the slow gamma, fast gamma, and SWR bands, but also for two control bands not necessarily related to cognition. While there were no main effects on Volume, there was an age and drug interaction seen for theta Volume that matched effects on theta event rate assessed through Morlet wavelets. Reanalysis through Morlet wavelets also uncovered maze location effects on average power in the slow and fast gamma range that were not replicated by DMDC. When data were collapsed across age, drug, location, and alternation groups, main effects of filter type on Dimension and Volume emerged. Thus, while DMDC resolves different levels of complexity when applied to different oscillation bands of the same signal, this approach may serve as a complementary means to better understand the roles of various LFP oscillation bands in brain development and spatial cognition.

## Methods

### Electrophysiological recordings

**Fig. 1:**
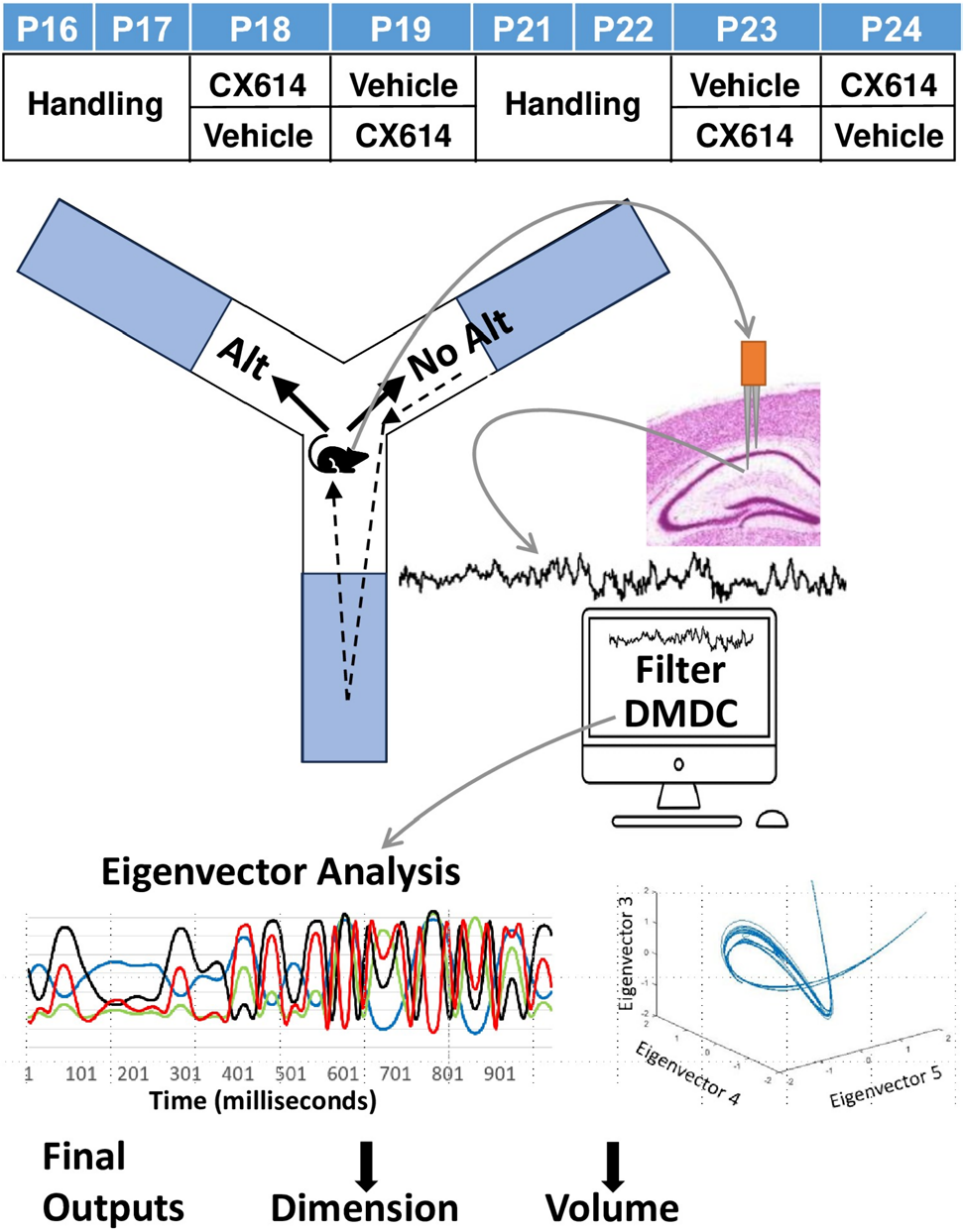
Schematic of data collection and analyses. LFP signals were continuously recorded as juvenile rats treated with vehicle or a positive AMPAR modulator (CX614, 2.5 mg/kg) freely navigated a symmetrical Y-maze (top left). Coronal image (taken from the Harvard Medical School, High Resolution Rat Brain Atlas) shows the recording location and a representative unfiltered LFP signal (top right). DMDC decomposes the filtered signal into a series of eigenvectors and compares values between eigenvectors across time (bottom left) and across each other to determine the shape of the attractor (bottom right) and related Dimension and Volume values

DMDC was applied to LFP recordings collected by McHail and Dumas (2020). These LFPs were recorded from the stratum radiatum region of area CA1 of the dorsal hippocampus in juvenile rats freely exploring a Y-maze on postnatal days (P) 18–19 (“Younger”) and P22-23 (“Older”). Each subject was tested at the Older and Younger ages twice. (Fig. 1, top). The AMPAKINE drug, CX614 (2.5 mg/kg or 4 mL/kg), or vehicle alone (cyclodextrin) was administered thirty minutes before every behavior test in a counterbalanced fashion (drug-vehicle for Younger followed by vehicle-drug for Older or vehicle-drug for Younger followed by drug-vehicle for Older) and each exploration lasted for eight minutes.

### Definition of signal Epochs for DMDC analysis

We identified events where the animal was immobile near the center of the Y-maze just prior to making an arm selection and moving through the maze center (Fig. 1, left side unshaded maze regions). DMDC was executed on 2500 ms of the LFP just preceding the movement. Additionally, we identified similar 2500 ms periods of immobility in the outer locations of the arms as control epochs when the animal was not making an arm selection (Fig. 1, left side shaded maze regions). All analyses of LFPs were conducted with Matlab including the signal processing toolbox.

### Signal processing

From the original raw LFP signal, multiple filters were created to isolate signal components using standard filtering packages in Matlab and some custom scripts. Biologically relevant band pass filters included the theta (4–12 Hz), slow gamma (25–55 Hz), fast gamma (65–100 Hz), and sharp wave ripples (SWR, 140–200 Hz). Additionally, we included control conditions of 20 Hz high pass (HP), 100 Hz low pass (LP), 100 Hz HP, 100–135 Hz, and a combination 4–100 Hz and SWR band pass (4–100 Hz + SWR). When running multiple signals concurrently (4–100 + SWR), DMDC relies on the assumption that the signals are causally related to each other and both are measurements of the same underlying dynamic​.​ By running the same signal differently filtered, relationships between multiple filters can be identified that may not be detectable in the unfiltered signal or when a single filter is applied.

The DMDC algorithm was written in-house and was executed with fixed parameters for all the runs. The number of delay coordinates was set to 8 and the number of nearest neighbors was set at 32. These were selected primarily from trial and error. Parameter sets were validated as successful when the results were persistent across parameter variations and the algorithm consistently converged for all of the samples taken at the center of the maze. Any samples in center or outer arm locations that returned a volume greater than 250 (2 in the center; 43 in outer) were discarded as this would not allow for adequate measurement of the attractor given our sampling time.

The same 2500 millisecond epochs were convolved using modified code from Cohen (2014). This code uses a series of Morlet wavelets at 1 Hz intervals from 1 to 101 Hz with number of cycles set to seven (Cohen, 2014). To account for the 1/f relationship of frequency to power and minimize the impact of differences in recording conditions across trials, each frequency band was then individually z-scored within a trial. These waveforms were broken down into theta, slow gamma, and fast gamma frequency bands. We then calculated average power for each band and also counted the number of times power surpassed a z-scored absolute value power of 1.95 (5% significance level) to estimate event rate within each frequency band. Average power and event rate were compared across age, drug, location, and frequency bands.

### Statistical analyses

Three-way analyses of variance (ANOVAs) were conducted to compare Dimension and Volume across age, drug condition, and maze location within each filter range for biologically relevant and control frequency bands. A three-way ANOVA was also conducted to compare Dimension and Volume across age, drug condition, and alternation choice when animals were at the maze center, just prior to making an arm selection. Alternation occurs when the animal chooses the least recently visited arm and non-alternation occurs when the animal does not choose the least recently visited arm (Douglas et al., 1973; Dumas, 2004; Blair et al., 2013). Average power and event rate were compared across age, drug condition, and maze location by three-way ANOVA. Data were collapsed across age, drug, location, and alternation and compared across filter type by one-way ANOVA. Linear regressions were calculated for mean Dimension or Volume versus filter band central tendency or range (Nyquist frequency limit for filters with no upper bounds). Post hoc pairwise comparisons were made by Tukey honest significant difference (HSD) tests. All bar graphs display means and confidence intervals (set at 95%).

## Results

### DMDC detects effects of age on dimension of biologically relevant band pass filtered hippocampal LFPs

**Fig. 2:**
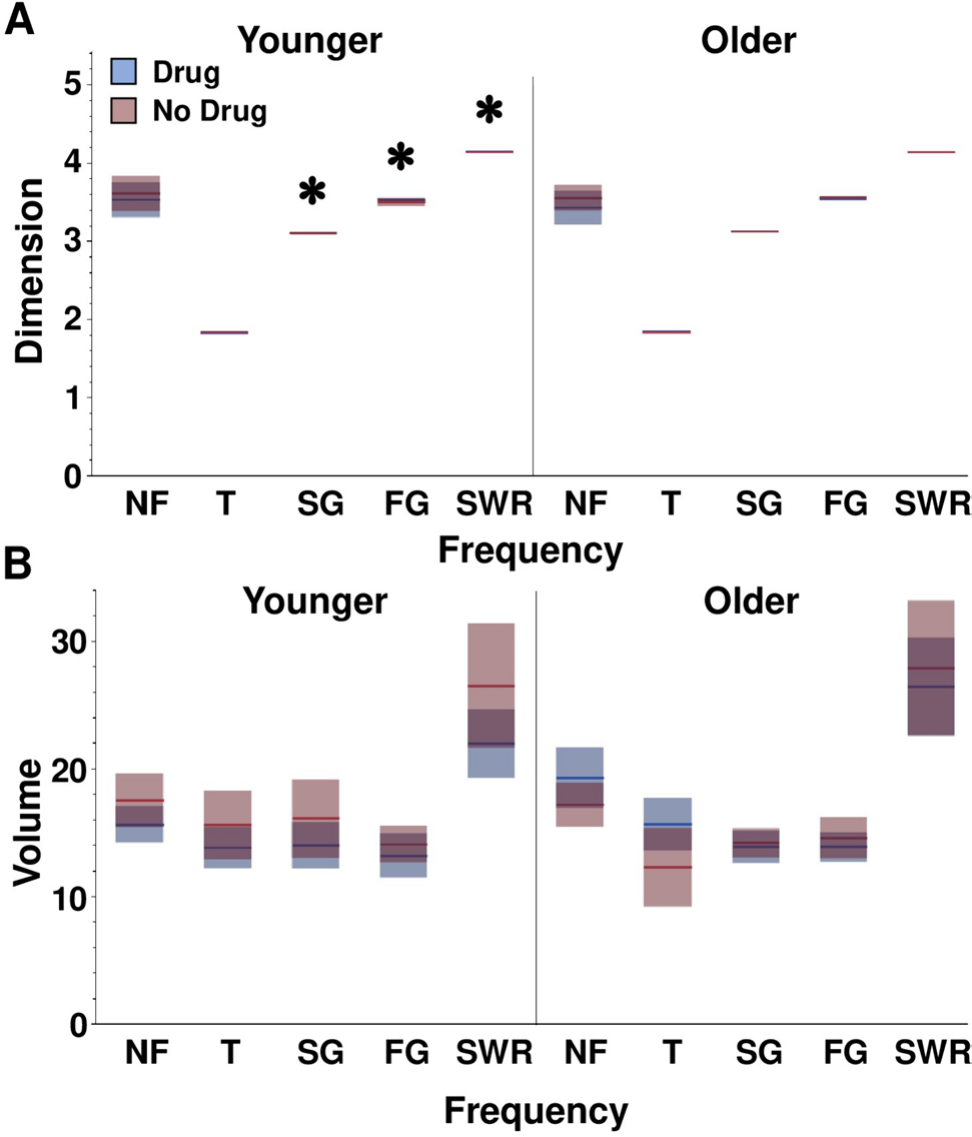
Dimension and Volume outcomes for biologically relevant oscillation frequency groups and the no filter control group separated by age and drug conditions. **A** Means and confidence intervals for Dimension across frequency groups. LFP signals from Older animals exhibited higher Dimension of slow gamma, fast gamma, and SWR filters compared to Younger animals. * represents a main effect of age. **B** Means and confidence intervals for Volume across frequency groups. Volume was unaffected by age, drug, or location conditions

We first applied DMDC to 2500 ms epochs of LFPs that were collected when subjects were at the center of the maze or in the outer portions of the arms. Prior to execution, signals were left unfiltered (No Filter) or band pass filtered for theta, slow gamma, fast gamma or SWRs. No main effects of age, drug, or location on Dimension or Volume were reported by DMDC for the unfiltered or theta filtered signal (Fig. 2; significant and non-significant statistics in Table 1). An age by drug interaction effect on Volume was observed for the theta band [F(1,124) = 3.4358, *p* = 0.0171]. Theta signals from Younger animals receiving CX614 exhibited decreased Volume compared to age match animals receiving vehicle (*p* < 0.05). Conversely, theta signals from Older animals receiving CX614 had increased Volume compared to age match animals receiving vehicle (*p* < 0.05).

**Table 1:** Summary of group effects for unfiltered, theta, slow gamma, fast gamma, and SWR signals. Statistically significant results are shown in bold red text. F is the ANOVA F ratio. P is the ANOVA probability value. NF = no filter. SG = slow gamma. FG = fast gamma. SWR = sharp wave ripple

**Dimension**	**NF**		**Theta**		**SG**		**FG**		**SWR**	
	**F**	**P**	**F**	**P**	**F**	**P**	**F**	**P**	**F**	**P**
Age	0.4202	0.5181	2.7314	0.1011	48.0816	**0.0001**	4.7450	**0.0314**	5.6270	**0.0193**
Drug	0.6151	0.4345	0.1810	0.6713	0.0087	0.9257	0.0792	0.7788	0.0166	0.8976
Age x drug	0.2611	0.6103	0.1679	0.6827	0.1341	0.7149	2.7381	0.1006	1.2517	0.2655
Location	0.0739	0.7862	0.0836	0.7730	0.0320	0.8582	0.1185	0.7312	0.6425	0.4244
Age x location	0.0515	0.8209	0.0521	0.8199	0.0121	0.9125	0.0663	0.7972	0.2522	0.6155
Drug x location	0.1572	0.6925	0.0007	0.9795	0.0532	0.8180	0.1039	0.7478	0.0212	0.8845
Age x drug x location	0.8695	0.3530	3.4358	0.0663	0.0616	0.8044	0.2048	0.6517	0.3862	0.5355
**Volume**	**F**	**P**	**F**	**P**	**F**	**P**	**F**	**P**	**F**	**P**
Age	2.6306	0.1075	0.0354	0.8511	0.6266	0.4302	0.3593	0.5501	1.5680	0.2130
Drug	0.0865	0.7692	0.8486	0.3588	1.0814	0.3005	0.8972	0.3455	1.0693	0.3032
Age x drug	3.8638	0.0517	5.847	**0.0171**	0.6889	0.4082	0.0007	0.9785	0.3923	0.5323
Location	0.9090	0.3423	0.3270	0.5685	0.1528	0.6966	1.1549	0.2827	0.9737	0.3258
Age x location	0.0080	0.9287	1.2159	0.2724	0.3757	0.5411	0.1405	0.7085	0.0631	0.8021
Drug x location	0.3476	0.5566	0.5963	0.4415	0.6889	0.4082	0.1069	0.7443	1.0668	0.3038
Age x drug x location	0.0057	0.9399	1.0931	0.2979	0.0158	0.9002	0.0343	0.8535	0.0217	0.8830

Dimension for slow gamma was higher for Older animals compared to Younger animals [main effect of age: F(1,124) = 48.0816, *p* < 0.0001] with no effect of drug or location. There were no effects of age, drug, or location on slow gamma Volume. In the fast gamma band, signals from Older animals showed higher Dimension values than signals from Younger animals [main effect of age: F(1,124) = 4.7450, *p* = 0.0314] with no effect of drug or location. No effect of age, drug, or location was reported for fast gamma Volume. In the SWR band, signals from Younger animals showed higher Dimension values than signals from Older animals [main effect of age: F(1,124) = 5.6270, *p* = 0.0193] with no effect of drug or location. No effect of age, drug, or location was reported for fast gamma Volume. Thus, barring one interaction effect, Volume was not sensitive to age, drug, or location and Dimension was affected by age only and limited to the slow gamma, fast gamma bands, and SWR bands.

### DMDC performed on control frequency bands produced effects that opposed outcomes from biologically relevant signal bands

Five control filters were applied to better understand the biological relevance of the initial bandpass analyses: **1)** high pass filter run above 20 Hz (20 Hz HP) to observe the slow gamma, fast gamma, and SWR filters together (excluding theta), **2)** low pass filter run below 100 Hz (100 Hz LP) to observe all of the previously studied spatial navigation filters together, **3)** high pass filter was executed above 100 Hz (100 Hz HP) to exclude the spatial navigation bands previously studied, **4)** bandpass from 100–135 Hz outside of the frequencies ascribed to spatial navigation bands to act as a band range control (similar to the band ranges for slow and fast gamma), and **5)** bandpass from 4–100 Hz in conjunction with the SWR band pass (SWR + 4–100 Hz). Previous research has shown that there may be correlation in activity of SWR and the gamma ranges (Carr et al., 2012; Pfeiffer & Foster, 2015).

**Fig. 3:**
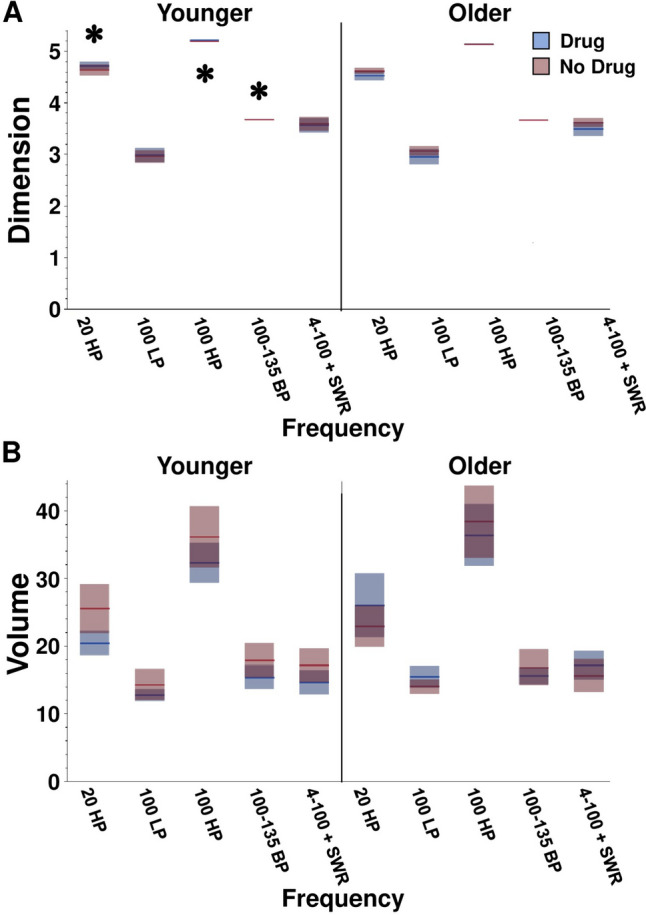
Dimension and Volume outcomes for control oscillation frequency groups separated by age and drug conditions. **A** Means and confidence intervals for Dimension across frequency groups. LFP signals from Younger animals exhibited higher Dimension of 20 Hz HP, 100 Hz HP, and 100–135 Hz filters compared to Older animals. # represents a main effect of location. **B** Means and confidence intervals for Volume across frequency groups. Volume was unaffected by age, drug, or location conditions. Signals recorded from Older animals contained more events in the theta band than signals recorded from Younger animals. * represents a main effect of age

Effects of age, drug, and location on Dimension or Volume for these control conditions were sparse (Fig. 3, significant and non-significant statistics in Table 2). No main effects of age, drug, or location on Dimension or Volume were reported by DMDC for the 100 Hz LP or the 4–100 + SWR group. For the 20 Hz HP, and in an opposing direction to results from individually analyzed slow gamma, fast gamma, and SWRs, Younger animals had a significantly higher Dimension than Older animals [main effect of age: F(1,124) = 5.4401, *p* = 0.0214] with no effect of drug or location. An age x drug interaction effect on Dimension opposite to that shown for the theta band was observed for the 20 Hz high pass filter [F(1,124) = 4.8780, *p* = 0.0291]. Dimension was increased for signals from Younger animals receiving CX614 compared to those receiving vehicle (*p* < 0.05) and decreased for signals from Older animals receiving CX614 compared to those receiving vehicle (*p* < 0.05). Volume was not impacted by age, drug, or location for the 20 Hz HP filtered signals. For the 100 Hz HP group, signals from Younger animals had a significantly higher Dimensions than Older animals [F(1,124) = 111.1, *p* < 0.0001] with no effect of drug or location. No main effect of age, drug, or location on Volume was observed for the 100 Hz HP filtered signals. For the 100 to 135 Hz bandpass, Dimension was higher in signals captured from Younger animals than it was in signals from Older animals [ main effect of age: F(1,124) = 27.8362, *p* < 0.0001], with no effect of drug or location. There was no main effect of age, drug, or location on Volume for the 100 to 135 Hz bandpass filtered signals. Thus, similar to the DMDC output for biologically relevant oscillation bands, Volume was not sensitive to age, drug, or location for any of the control filtered signals and Dimension was affected almost exclusively by age (barring one age by drug interaction). Interestingly, main and interaction effects observed in the control conditions were all opposite of those observed in the biologically relevant signals.

**Table 2:** Summary of comparative statistics for Dimension and Volume values extracted from differently filtered control LFPs. Statistically significant results are shown in bold red text. F is the ANOVA F ratio. P is the ANOVA probability value

Dimension	20 Hz HP		100 Hz LP		100 Hz HP		100–135 Hz		4–100 Hz + SWR	
	F	P	F	P	F	P	F	P	F	P
Age	5.4401	**0.0214**	0.4420	0.5075	111.12	** < 0.0001**	27.8362	** < 0.0001**	0.0707	0.7908
Drug	0.0006	0.9799	0.4876	0.4864	2.6472	0.1064	0.3246	0.5699	0.9502	0.3317
Age x drug	4.8780	**0.0291**	1.115	0.2939	2.9328	0.0894	0.1002	0.7522	1.0759	0.3017
Location	0.3452	0.5580	0.5395	0.4641	0.0149	0.8997	1.3949	0.2400	0.0307	0.8327
Age x location	0.0193	0.8899	0.0590	0.8086	0.0196	0.8889	0.5136	0.4750	0.0448	0.8327
Drug x location	0.0557	0.8138	0.0107	0.9177	0.0001	0.9934	0.0765	0.7825	0.3140	0.5763
Age x drug x location	0.2506	0.6176	0.0013	0.9718	0.0567	0.8122	0.0925	0.7616	1.0542	0.3066
Volume	F	P	F	P	F	P	F	P	F	P
Age	0.8213	0.3666	2.1751	0.1429	1.8044	0.1818	0.0694	0.7927	0.0707	0.7908
Drug	0.3986	0.5290	0.0069	0.9337	1.6460	0.2020	2.2187	0.1390	0.9502	0.3317
Age x drug	3.1194	0.0800	3.9053	0.0505	0.0735	0.7868	0.2910	0.5906	1.0759	0.3017
Location	2.0886	0.1511	0.0975	0.7544	0.0517	0.8206	0.8321	0.3635	0.0307	0.8612
Age x location	0.0936	0.7601	0.0001	0.9905	0.0006	0.9807	0.0867	0.7689	0.0448	0.8327
Drug x location	0.0184	0.8923	0.2095	0.6480	0.0166	0.8978	0.3260	0.5691	0.3140	0.5763
Age x drug x location	2.1128	0.1487	0.0707	0.7908	0.1461	0.7030	0.0008	0.9776	1.0542	0.3066

### More traditional power analyses revealed age and location effects for biologically relevant oscillation bands that contrasted DMDC outcomes

**Fig. 4:**
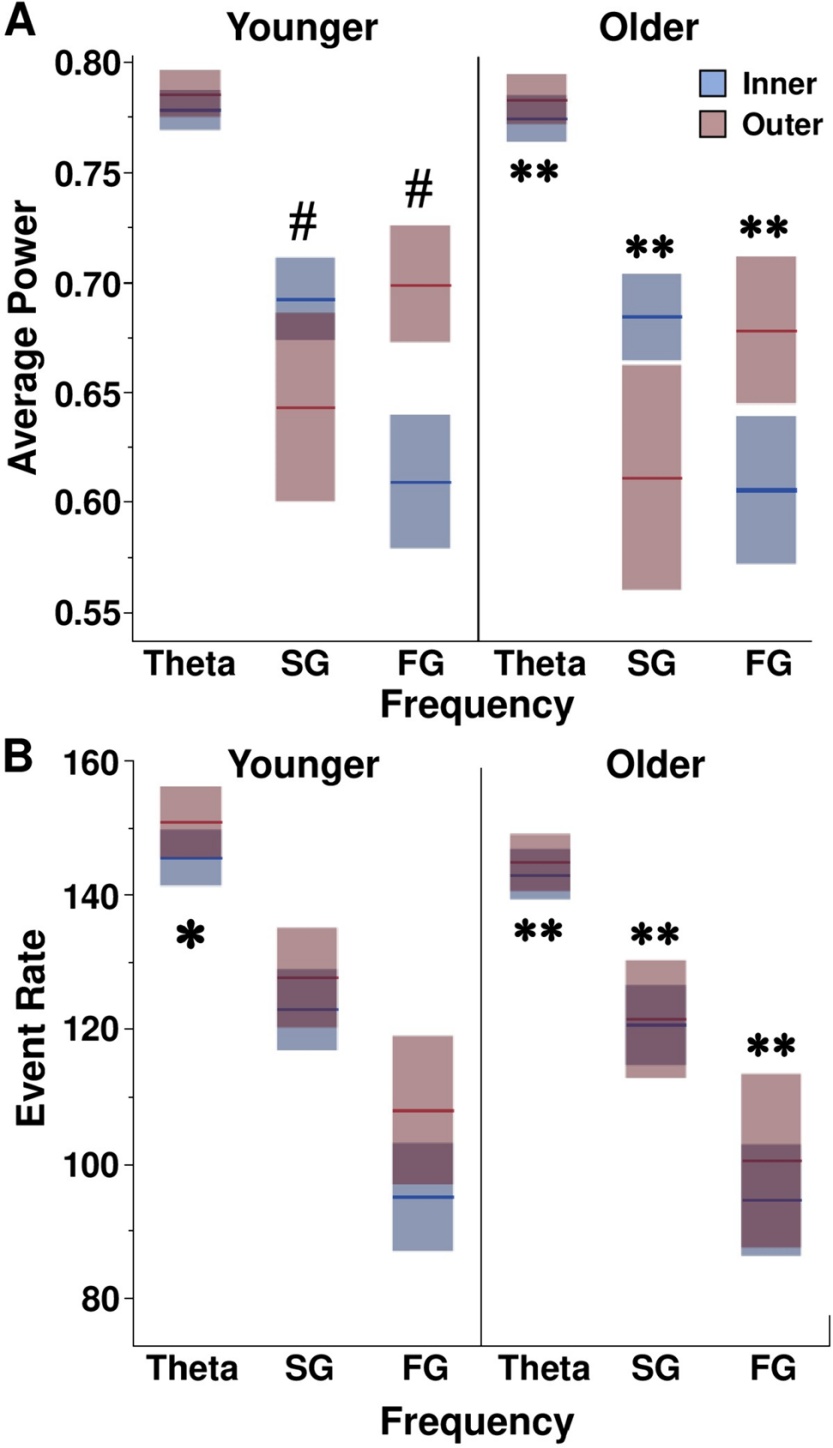
Mean power and event rate outcomes for biologically relevant oscillation frequency groups separated by age and location conditions. **A** Means and confidence intervals for average power across frequency groups. Slow gamma power was greater in LFP signals recorded in the inner portion of the arms. Fast gamma power was greater in signals recorded in the outer portions of the maze arms. * represents a main effect of location. **B** Means and confidence intervals for event rate across frequency groups. * represents a significant main effect of age. # represents a significant main effect of location. ** represents significant post hoc results for filter type

To compare DMDC output to more traditional power analyses, we calculated average power (Fig. 4A) and event rate (Fig. 4B) across biologically relevant oscillation frequency bands and compared across frequency ranges, age, drug, and location groups. The SWR band was not included because the wavelet calculation spans from 1 to 100 Hz to align with the analyses performed in McHail and Dumas (2020). Average power differed across frequency range groups [F(2,249) = 98.4353, *p* < 0.0001]. Average power was higher in theta than slow gamma (*p* < 0.05) or fast gamma (*p* < 0.05) and average power in slow gamma was higher than fast gamma (*p* < 0.05). This outcome was expected since the 1/f relationship of frequency and power in natural signals usually results in higher power at lower frequencies. There was a location effect for both slow [F(1,82) = 12.4241, *p* = 0.0007] and fast gamma bands [F(1,82) = 29.0083, *p* < 0.0001]. Slow gamma power was increased in animals in the center of the maze compared to animals in the outer portion of the maze (*p* < 0.05), while fast gamma power was decreased for animals in the center of the maze compared to animals in the outer portion of the maze (*p* < 0.05). There was no main effect of age, drug, or location on average power in the theta band. There were no interaction effects on average power seen amongst the three filters.

There was a significant difference in event rate between the three frequency ranges [F(2, 249) = 158.6187, *p* < 0.0001] (Fig. 4B). The theta frequency range had more events than the slow gamma (*p* < 0.05) or fast gamma (*p* < 0.05) and slow gamma had more events than fast gamma (*p* < 0.05). Signals recorded from Older animals contained more events in the theta band [main effect of age: F(1,82) = 8.8462, *p* = 0.0039], but there was no effect of drug or location. There was also an interaction effect of age x drug on theta event rate [F(1,82) = 4.4891, *p* = 0.0374]. Signals from Older animals receiving CX614 contained more events in the theta band compared to animals receiving vehicle (*p* < 0.05), while Younger animals receiving CX614 had a lower event rate than vehicle counterparts (*p* < 0.05). There was no main effect of age, drug, or location on slow or fast gamma event rate. Thus, mean power and event rate results from more traditional analyses identify location but not age effects better than DMDC.

### DMDC does not differentiate between alternation and non-alternation trials

We next determined if DMDC could distinguish between trials in which the animal subsequently alternated or did not alternate in its maze arm selection. Signals from outer arm regions were omitted and signals collected when the animal was facing the maze center were compared across age, drug, and alternation versus non-alternation categories for all filter types applied to the prior location comparisons. DMDC did not report any effect of alternation versus non-alternation on Dimension or Volume in the unfiltered, theta, slow gamma, fast gamma, SWR, 20 Hz High Pass, 100 Hz LP, 100 Hz HP, 100–135 Hz, or 4–100 Hz + SWR conditions (Table 3). Combined with the initial DMDC outcomes, it appears that DMDC better identifies more static or holistic features of the LFP signals (age) than short-term signal dynamics (location, alternation).

**Table 3:** Summary of comparative statistics for average power and event rate for biologically relevant LFP signals. Statistically significant results are shown in bold red text. F is the ANOVA F ratio. P is the ANOVA probability value. SG = slow gamma. FG = fast gamma

**Power**	**Theta**		**SG**		**FG**	
	**F**	**P**	**F**	**P**	**F**	**P**
Age	0.3961	0.531	1.2566	0.2658	0.6980	0.4061
Drug	0.0158	0.9003	0.3703	0.5447	0.0797	0.7785
Drug x age	0.0146	0.9041	0.0016	0.9683	0.0997	0.7531
Location	2.388	0.1264	12.4241	0.0007	29.0083	< 0.0001
Age x location	0.0261	0.8721	0.3752	0.5420	0.2472	0.6205
Drug x location	1.2691	0.2635	1.6480	0.2031	2.3177	0.1321
Drug x age x location	0.6489	0.423	0.2968	0.5876	0.1589	0.6913

**Event Rate**	**Theta**		**SG**		**FG**	
	**F**	**P**	**F**	**P**	**F**	**P**
Age	4.4891	0.0039	1.4513	0.2321	0.6573	0.4201
Drug	0.0776	0.7814	0.1130	0.7367	0.0003	0.9868
Drug x age	8.8462	0.0374	0.0859	0.7702	0.1472	0.7023
Location	3.1809	0.0785	0.6359	0.4277	3.54282	0.0634
Location	3.1809	0.0785	0.6359	0.4277	3.54282	0.0634
Drug x location	1.7549	0.1892	1.6452	0.2035	2.4954	0.1183
Drug x age x location	0.1920	0.6625	0.2756	0.6011	0.0001	0.9905

### DMDC reveals different dimensions and volumes for different LFP filters

Since different filtering types revealed differences in age and drug effects, we examined the contribution of filtering itself to the DMDC outcomes by directly comparing across filter types (unfiltered, theta, slow gamma, fast gamma, SWR, 20 Hz HP, 100 Hz LP, 100 Hz HP, 100–135 Hz, and 4–100 Hz + SWR) after collapsing across age, drug, location, and alternation variables. DMDC found multiple significant effects of filter type on Dimension [F(9,1250) = 1646.967, *p* < 0.0001] (Table 4). The only filter groups that did not differ were the no filter group compared to the fast gamma or 4–100 + SWR group. Also, the fast gamma group did not differ from SWR and the 100–135 Hz group did not differ from 4–100 + SWR.

**Table 4:** Tukey tests results comparing Dimension values across filters. * represents a *p*-value equal to or less than 0.05, ** represents a *p*-value less than 0.01, *** represents a *p*-value less than 0.0001, and n.s. represents any p-value greater than or equal to 0.05. NF = no filter. SG = slow gamma. FG = fast gamma. SWR = sharp wave ripple

	**NF**	**Theta**	**SG**	**FG**	**SWR**	**20 HP**	**100 LP**	**100 HP**	**100–135**	**4–100 + SWR**
**NF**		***	***	n.s	***	***	***	***	**	n.s
**Theta**	***		***	***	***	***	***	***	***	***
**SG**	***	***		***	***	***	**	***	***	**
**FG**	n.s	***	***		n.s	***	***	***	**	***
**SWR**	***	***	***	n.s		***	***	***	**	***
**20 HP**	***	***	***	***	***		***	***	***	***
**100 LP**	***	***	**	***	***	***		***	***	***
**100 HP**	***	***	***	***	***	***	***		***	***
**100–135**	***	***	***	**	***	***	***	***		*
** 4–100 + SWR**	n.s	***	**	***	***	***	***	***	***	

**Fig. 5:**
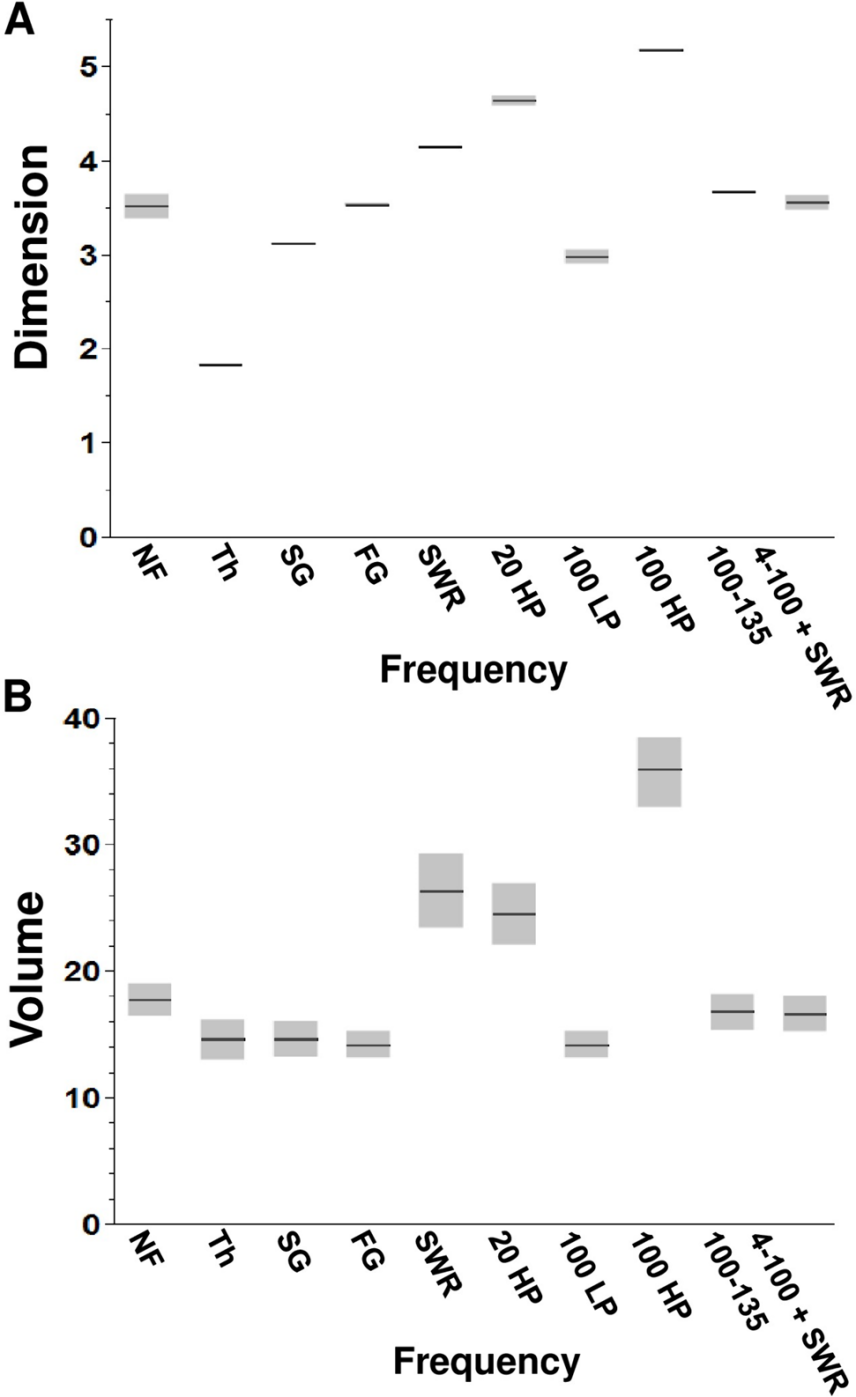
Dimension and Volume outcomes across oscillation frequency groups collapsed across age, drug, location, and alternation conditions. **A** Means and confidence intervals for Dimension across frequency groups. **B** Means and confidence intervals for Volume across frequency groups

DMDC also reported a main effect of filter type on Volume [F(9,1250) = 107.7878, *p* < 0.0001] (Table 5). Significant pairwise comparisons were more limited than for Dimension. Volume for the 20 Hz HP and SWR filter groups differed most frequently from the other filter groups (*p* < 0.0001 compared to all other filter groups but not each other). Volume for the slow gamma and fast gamma groups also differed from the 100 Hz HP group (each at *p* < 0.0001) (Fig. 5B) (Tables 4 and 5).

**Table 5:** Tukey tests results comparing Volume values across filters. * represents a *p*-value equal to or less than 0.05, ** represents a *p*-value less than 0.01, *** represents a p-value less than 0.0001, and n.s. represents any p-value greater than or equal to 0.05. NF = no filter. SG = slow gamma. FG = fast gamma. SWR = sharp wave ripple

	**NF**	**Theta**	**SG**	**FG**	**SWR**	**20 HP**	**100 LP**	**100 HP**	**100–135**	**4–100 + SWR**
**NF**		*	n.s	**	***	***	*	***	n.s	n.s
**Theta**	*		n.s	n.s	***	***	n.s	***	n.s	n.s
**SG**	n.s	n.s		n.s	***	***	n.s	***	n.s	n.s
**FG**	**	n.s	n.s		***	***	n.s	***	n.s	n.s
**SWR**	**	***	***	***		n.s	***	***	***	***
**20 HP**	***	***	***	***	n.s		***	***	***	***
**100 LP**	*	n.s	n.s	n.s	***	***		***	n.s	n.s
**100 HP**	***	***	***	***	***	***	***		***	***
**100–135**	n.s	n.s	n.s	n.s	***	***	n.s	***		n.s
**4–100 + SWR**	*	n.s	n.s	n.s	***	***	***	***	n.s

**Fig. 6:**
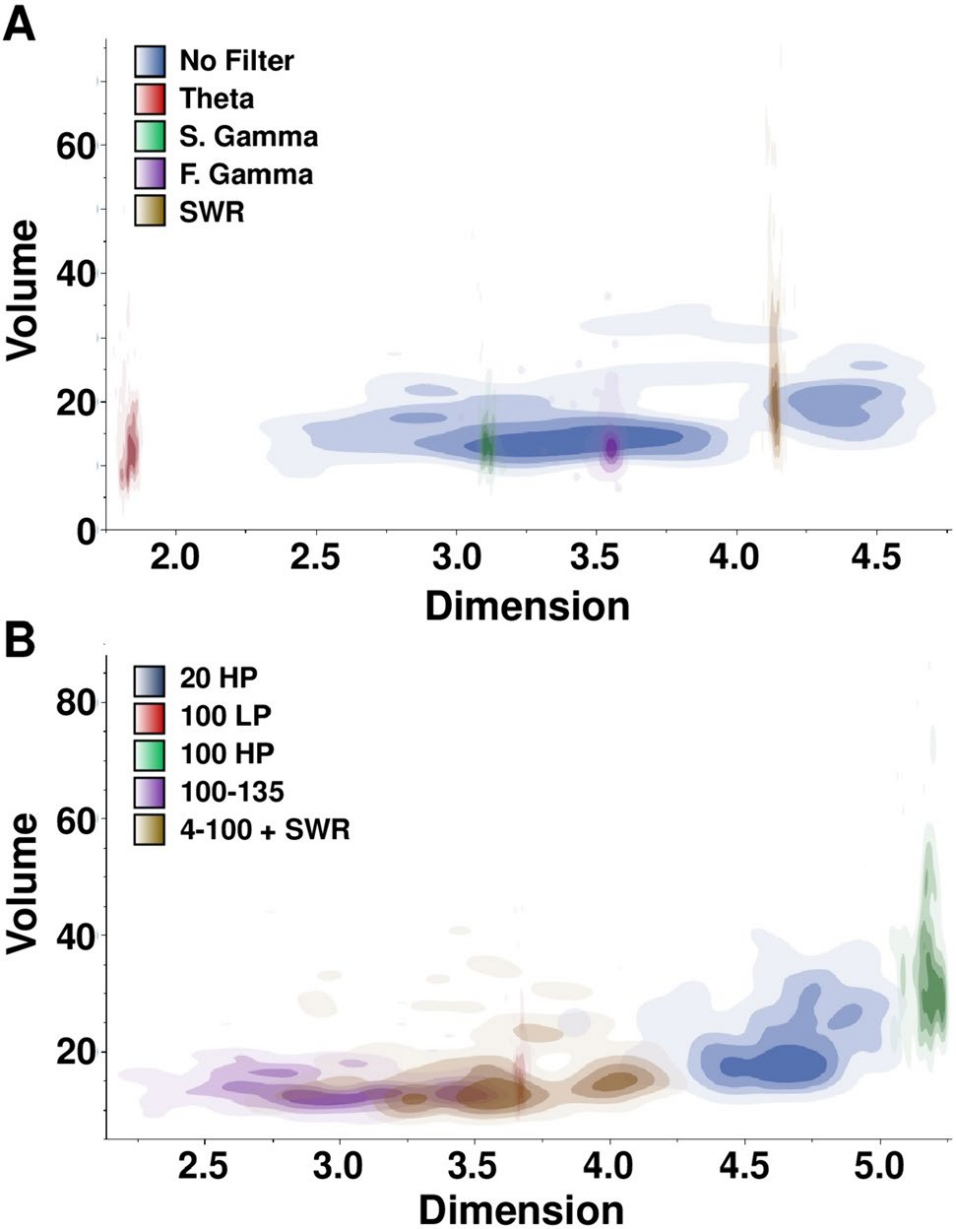
Dimension plotted against Volume when collapsed across age, drug, location, and alternation conditions. **A** Volume versus Dimension for biologically relevant frequency groups and the no filter control group. **B** Volume versus Dimension for control frequency groups

When Volume was plotted against Dimension (Fig. 6), filters that included higher frequency ranges, like the 20 and 100 Hz high pass, the SWR band pass, appeared to have the highest Dimension while filters that included the lowest frequency ranges, such as theta, seemed to have the lowest Dimension (Fig. 6A).

**Fig. 7:**
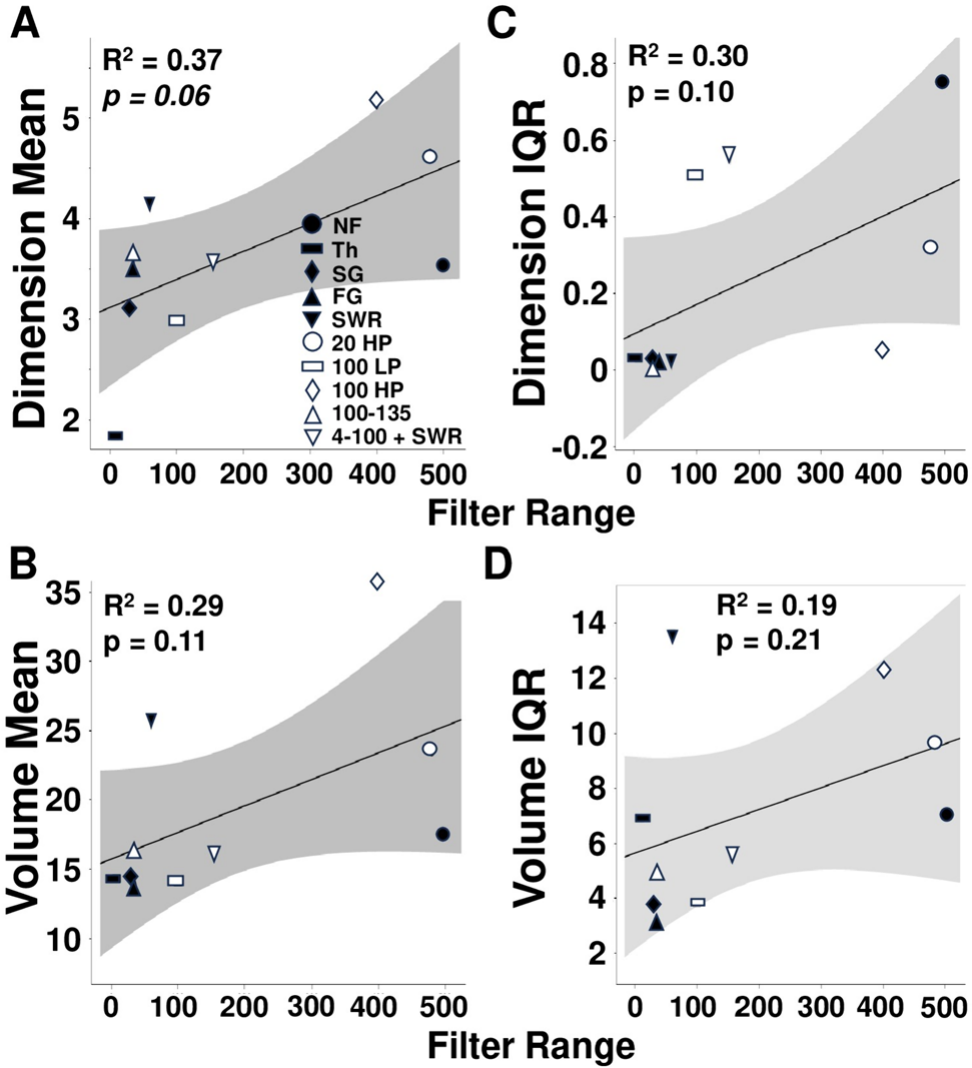
Regression calculation and 95% confidence limits for filter range versus mean Dimension or Volume or variability in Dimension or Volume. **A** Linear regression for Dimension Mean plotted against filter range. **B** Linear regression for Volume Mean plotted against filter range. **C** Linear regression for Dimension interquartile range (IQR) plotted against filter range. **D** Linear regression for Volume interquartile range (IQR) plotted against filter range

This trend for a relationship between frequency range and Dimension was apparent for Volume as well (Fig. 6B), though linear regressions for central tendency of the filter group [Dimension: R^2^ = 0.365, t(9) = 2.15, *p* = 0.641; Volume: R^2^ = 0.287, t(9) = 1.79, *p* = 0.1105] or variance of the filter group [Dimension: R^2^ = 0.296, t(9) = 1.83, *p* = 0.1042; Volume: R^2^ = 0.190, t(9) = 1.37, *p* = 0.2079] versus Dimension or Volume were not significant (Fig. 7).

## Discussion

Overall, DMDC appears to provide a relatively conservative view of differences in LFP signals based on age, drug treatment, spatial location, or alternation decision during free exploration in a Y-maze. Dimension was affected by age (increased in slow and fast gamma bands in Older subjects), but not drug condition, location, or alternation decision in biologically relevant oscillation bands. Volume was not sensitive to any of these independent variables (except one age by drug interaction in the theta band). Slow and fast gamma oscillations are mediated by separate synaptic inputs into area CA1 with slow gamma more closely tied to input from area CA3 and fast gamma linked to increases in activity from the entorhinal cortex (Charpak et al., 1995; Colgin et al., 2009). As such, increased Dimension in the slow and fast gamma bands might reflect continued maturation of CA3 and TA inputs into area CA1 across the ages tested. Some age effects on Dimension present in control filter conditions may reflect age effects in the original bandpass set (age effect on Dimension for 20 Hz HP, age x drug interaction for Dimension of 20 Hz HP). Age effects were opposite in direction of effects seen in the biologically relevant bands possibly due to combining biologically relevant frequency bands with each other or with intervening frequency bands (ex. 20 Hz HP or 100 Hz HP). Lack of effects for various control frequency ranges strengthen the notion that, when LFP signals are appropriately filtered, DMDC identifies network oscillation frequencies that are altered during postnatal development and that may not be apparent using traditional analyses.

Dimension results partially conform with outcomes from more traditional analyses previously applied (McHail & Dumas, 2020) in that increased Dimension for fast gamma recorded in Older subjects parallels an age-related increase in fast gamma event rate determined by Morlet wavelets. Additionally, an age by drug interaction existed for theta Volume and theta event rate following reanalysis with traditional methods. Theta Volume and event rate decreased after administration of CX614 in the Younger group while Volume and event rate increased after administration of CX614 in the Older group. However, reanalysis by more traditional approaches also revealed location effects that were not captured by DMDC and produced opposing effects of location for slow gamma (higher at choice points) and fast gamma power (lower at choice points) and increased theta event rate in Older animals. During periods of immobility prior to movement to a known goal location, slow gamma power in hippocampal area CA1 increases (Leung, 1998) along with the frequency of SWRs (Buzsáki et al., 2003; Pfeiffer & Foster, 2015). In contrast, fast gamma power increases more during spatial navigation (Cabral et al., 2014). Thus, it might be expected that slow, but not fast gamma power was increased at the Y-maze choice points as we observed. Differences between prior and current results for average power and event rate may stem from differences in temporal ranges used for analyses or different selection criteria for samples. In the previous analyses, the entire trial was previewed, regardless of animal state or location, and then samples that reached event threshold were analyzed (McHail & Dumas, 2020). Instead, we categorized by position, independent of signal quality, and then performed our analyses.

Dimension refers to the minimum number of dimensions across which the signal must be projected so as to eliminate any overlapping points in the attractor. Although higher dimensions provide more degrees of freedom, they may or may not be more “complex”. Thus, increased Dimension in the slow gamma, fast gamma, and SWR bands of Older subjects may suggest a greater number of dynamic states the hippocampus is able to achieve within these oscillation bands with increasing age. Alternatively, increased Dimension in slow and fast gamma oscillations with increasing age may reflect a more prominent higher dimension state or low and high state dimensional embedding. Dimension does not align with behavioral attributes (location, alternation choice) supporting the idea that the manifolds produced by DMDC best reflect more stationary aspects of the LFP signal and less so for dynamics that vary within the signal epoch. However, the finding that DMDC identified age effects on Dimension for slow gamma, fast gamma, and SWR oscillations but not for the unfiltered signal argues that DMDC views these oscillation bands differently when isolated or when embedded in a more complex signal and may require more data to be accurately disentangled when unfiltered. A better understanding of the LFP signal parameters that influence Dimension and Volume may be gleaned by 1) applying DMDC to artificial LFP signals of known properties and measuring the sensitivity to various signal components and 2) comparing DMDC outcomes to results from similar unsupervised machine learning approaches, such as hidden Markov models (Masaracchia et al., 2023).

The filter conditions that tended to yield the highest Dimension scores were the higher frequency filters. When combined with lower frequency oscillations, it might be expected that high frequency components enable encoding of more detailed information over a fixed epoch. This is consistent with the fact that slow gamma oscillations are often embedded in and modulated by theta oscillations (Lisman & Jensen, 2013). Thus, the greater the coupling between the lower and higher frequency signals, the more influence the higher frequency signal may have on the Dimension output. Alternatively, the increase in Dimension with increasing frequency band could also indicate that separate attractors underlie the activity in lower and higher frequency ranges that then combine to produce the filtering effect.

Interpretation of Volume may be more straightforward. The Volume that DMDC reports is the average length that the attractor takes to complete an average period. For example, if within a sample DMDC detects multiple attractor cycles, the algorithm averages the lengths of these cycles to produce a Volume score. As this is a one-dimensional temporal signal, Volume is correlated to the average period of the events within the signal, not necessarily the average frequency detected within a signal since events can occur over a number of oscillations. Given that Volume tends to increase with the frequency of oscillations in these data adds credence to the notion that higher frequencies encode more complex events.

It should be noted that the variability in Dimension and Volume values within groups in the current is much lower than would be expected given results from prior applications of DMDC (Berry et al., 2013). To explain the reduced error in the current study, we should consider how DMDC models an attractor given the signal input. DMDC compares multiple hypothetical attractor loops against each other to determine a mean Dimension and Volume for the attractor of the signal. For example, for a 2500 ms signal, if DMDC models an attractor with a Volume of 100 ms then it will attempt to determine the dimensionality of the 25 periods of the attractor throughout the signal. If Volume is short relative to the duration of the signal, it will return a fairly consistent measurement when it does indeed capture an attractor, as it has seen sufficient cycles be able to produce a reliable estimate. Thus, the low degree of variance in Volume scores is supportive of accurate identification of attractors.

Dimension scores were sometimes higher than expected given the length of the input signal (Berry et al., 2013). While it is possible that this could indicate complex processes occurring in these frequency ranges, it is more likely that DMDC is capturing strong uncorrelated noise where the random variations in the signal fill the embedding space and produces a higher Dimension. This latter scenario appears more likely, as a reliable characterization of high dimensional signals (4 +) from one dimensional data typically requires sample times that contain hundreds of periods of the dynamics. Although our segments are too short to properly interrogate high dimensional dynamics, the more fundamental issue lies in the fact that the neuronal state may not persist long enough to acquire the requisite observations. It should still be possible to investigate these higher dimensional states by concatenating a large number of observations of the same state. However this procedure requires the initial segregation of the data into different dynamics, which presents its own issues. Moreover, multiple electrodes could be used to capture sufficient data over short epochs to resolve higher dimensions. Regardless, we believe we have shown that DMDC reveals signal properties that traditional frequency analyses do not and can serve as a complimentary tool to use in conjunction with other forms of LFP frequency analyses.

While we used DMDC to combine two different filter bands of the same signal (4–100 Hz + SWR), when applied to separate recordings from different brain regions or hippocampal subregions, this approach might reveal interstructural attractor states (Ventrucci et al., 2014). For instance, area CA1, the subiculum, and the entorhinal cortex (EC) work together to both retrieve and consolidate spatial memories (Chrobak & Buzáki, 1996; Colgin, 2016; Joo & Frank, 2018). In an orthograde direction, area CA1 projects separately to the subiculum and the EC and the subiculum projects to the EC (Naber et al., 2001). Antidromically, the subiculum projects to area CA1. These pathways are likely differentially active both with respect to cognitive state. However, it is unclear how and when these structures are interacting to retrieve or consolidate spatial memories in awake subjects. Thus, separating LFP epochs corresponding to different stages of training or memory retrieval trials and analyzing the signals from these three sites concurrently via DMDC might reveal Dimension and/or Volume outputs that better define different cognitive states.

## Data Availability

Raw and analyzed data are available upon request.
